# Regression of orthotopic neuroblastoma in mice by targeting the endothelial and tumor cell compartments

**DOI:** 10.1186/1479-5876-7-16

**Published:** 2009-03-12

**Authors:** Dieter Fuchs, Rolf Christofferson, Mats Stridsberg, Elin Lindhagen, Faranak Azarbayjani

**Affiliations:** 1 Department of Medical Cell Biology, Uppsala University, 75123 Uppsala, Sweden; 2 Department of Woman and Child Health, Uppsala University Hospital, 75185 Uppsala, Sweden; 3 Department of Medical Sciences, Uppsala University Hospital, 75185 Uppsala, Sweden

## Abstract

**Background:**

High-risk neuroblastoma has an overall five-year survival of less than 40%, indicating a need for new treatment strategies such as angiogenesis inhibition. Recent studies have shown that chemotherapeutic drugs can inhibit angiogenesis if administered in a continuous schedule. The aim of this study was primarily to characterize tumor spread in an orthotopic, metastatic model for aggressive, MYCN-amplified neuroblastoma and secondarily to study the effects of daily administration of the chemotherapeutic agent CHS 828 on tumor angiogenesis, tumor growth, and spread.

**Methods:**

MYCN-amplified human neuroblastoma cells (IMR-32, 2 × 10^6^) were injected into the left adrenal gland in SCID mice through a flank incision. Nine weeks later, a new laparotomy was performed to confirm tumor establishment and to estimate tumor volume. Animals were randomized to either treatment with CHS 828 (20 mg/kg/day; p.o.) or vehicle control. Differences between groups in tumor volume were analyzed by Mann-Whitney *U *test and in metastatic spread using Fisher's exact test. Differences with p < 0.05 were considered statistically significant.

**Results:**

The orthotopic model resembled clinical neuroblastoma in respect to tumor site, growth and spread. Treatment with CHS 828 resulted in tumor regression (p < 0.001) and reduction in viable tumor fraction (p < 0.001) and metastatic spread (p < 0.05) in correlation with reduced plasma levels of the putative tumor marker chromogranin A (p < 0.001). These effects were due to increased tumor cell death and reduced angiogenesis. No treatment-related toxicities were observed.

**Conclusion:**

The metastatic animal model in this study resembled clinical neuroblastoma and is therefore clinically relevant for examining new treatment strategies for this malignancy. Our results indicate that daily scheduling of CHS 828 may be beneficial in treating patients with high-risk neuroblastoma.

## Background

Neuroblastoma (NB) is the most common extracranial solid tumor of childhood. High-risk NB has a long-term survival rate of less than 40% despite intensive treatment protocols involving high-dose chemotherapy, usually with bone marrow rescue, aggressive surgery, and radiotherapy [1,2]. Therefore, new treatment strategies, evaluated in clinically relevant, reliable, and reproducible animal models, are needed for this malignancy.

Angiogenesis inhibition is a novel treatment strategy, where the formation of new blood vessels is inhibited, thereby reducing both the metabolic exchange of the tumor and its vascular access for metastatic spread. In NB, a high tumor angiogenesis correlates with metastatic disease and poor outcome [3]. Furthermore, increased microvascular proliferation has recently been shown to correlate with poor survival in children with NB [4]. There are many ways for angiogenesis inhibition, e.g. specific inhibition of an angiogenic growth factor. In s.c. models for NB, this approach resulted in a significantly reduced tumor growth rate [5,6]. Another way for angiogenesis inhibition is based on modified schedules and doses of chemotherapeutic drugs, namely, switching from the current maximum tolerable dose (MTD) to a continuous dosing scheme [7]. Even though endothelial cells are damaged by MTD, the beneficial antiangiogenic effects of MTD schedules are compromised by treatment breaks between cycles. These breaks are required for patient recovery but allow endothelial cell repair and regrowth [8,9]. Chemotherapy given at frequent intervals without extended rest periods, has been shown to target endothelial cells and tumor vessels *in vivo *[10]. The benefits of continuous therapy, e.g. reduced host toxicity together with continuous drug exposure resulting in a sustained antiangiogenic effect, are investigated in a number of clinical trials [11].

The chemotherapeutic drug CHS 828 is a pyridylguanidine that potently inhibits nicotinamide phosphoribosyl transferase (NAMPT) in a time dependent manner [12,13]. NAMPT is an enzyme involved in the biosynthesis of oxidized nicotinamide adenine dinucleotide (NAD^+^). In eukaryotic cells NAD^+ ^has been shown to play a pivotal role as an essential coenzyme/transmitter molecule for the generation of ATP. Due to the higher proliferation rate, cancer cells demand higher ATP synthesis and therefore have higher turnover of NAD^+ ^and an upregulated NAMPT enzyme to meet this energy demand. In fact, NAMPT inhibition with CHS 828 has shown significant antitumor activity in many preclinical *in vitro *and *in vivo *models [14-17]. In clinical phase I studies conducted with CHS 828, doses up to 500 mg were administered to patients. Based on the observed dose limiting toxicities at 500 mg (228 mg/m^2^), Ravaud *et al*. suggested administration of 420 mg CHS 828 every 3 weeks for clinical phase II studies [18] whereas the results of another clinical phase I study recommended more frequent administration at 20 mg once a day for 5 days in cycles of 28 days duration [19].

In preclinical studies in mice, CHS 828 could reduce growth of s.c. NB without any signs of toxicity [17]. In order to investigate this finding in a clinically more relevant setting, we developed and characterized a relevant orthotopic mouse models for high-risk NB. Generally, orthotopic tumor models resemble clinical disseminated disease more closely and have a more realistic tumor-host interaction than heterotopic, s.c. models. To be able to evaluate and to make a direct comparison between these models in treating NB, mice bearing orthotopic tumors were treated with the same dose and route of administration as in [17].

We found that the orthotopic growth and spread of NB cells in SCID mice resembled the patterns observed in high-risk NB patients. Daily oral administration of a non-toxic dose of CHS 828 to the host animal induced tumor regression and reduced bone marrow and liver metastases by a dual mechanism of action, restraining growth of both tumor cells and tumor vasculature.

## Methods

### CHS 828

The chemotherapeutic drug CHS 828 (*N*-(6-chlorophenoxyhexyl)-*N*'-cyano-*N*"-4-pyridylguanidine) was supplied by LEO Pharma (Ballerup, Denmark). For *in vitro *use, CHS 828 was dissolved to 5 mM in dimethyl sulfoxide (DMSO) (Merck, Darmstadt, Germany) and further diluted in serum-free culture medium. For the *in vivo *study, the drug was suspended in peanut oil (5 μg/μl) at least once a week and stored at 4–8°C.

### Cells

The human NB cell line IMR-32 (ATCC, Rockville, MD), isolated from an abdominal NB in a 13-month-old boy, is MYCN amplified and has a 1p deletion and a 47 + XY karyotype [20]. SH-SY5Y (kindly provided by Dr. June Biedler, The Memorial Sloan-Kettering Cancer Centre, NY) was derived from a poorly differentiated, non-MYCN-amplified human NB [21]. SK-N-SH, a kind gift of Dr. Fredrik Hedborg, Uppsala University, Sweden, was isolated from a bone marrow metastasis of a 4 year old female NB patient. Cells were cultured as described previously [5]. Non-essential amino acids (Sigma Chemical Co., St. Louis, MO) were added to IMR-32 cells. Human foreskin fibroblasts (CCD-1064SK, a kind gift of Dr. Magnus Essand, Uppsala University, Sweden) were cultured under the same conditions as SH-SY5Y [5]. Immortalized bovine endothelial cells (hTERT-BCE [22], a kind gift from Dr. Yihai Cao, Karolinska Institute, Stockholm, Sweden), were cultured as described previously [22].

All cells tested negative for mycoplasms and were grown in humidified air (95%) and 5% CO_2 _at 37°C. All *in vitro *experiments were performed under optimal culture conditions (i.e. with serum).

### Fluorometric microculture cytotoxicity assay

Drug cytotoxicity was determined using the fluorometric microculture cytotoxicity assay (FMCA) method [23]. Briefly, CHS 828 stock solution, dissolved to 5 mM in DMSO, was diluted in medium to final concentrations ranging from 0.1 nM to 10 μM. Triplicates of drug solutions (10 × final concentration; 20 μl) were added to v-bottomed 96-well microtiter plates (Nunc, Roskilde, Denmark). NB cells (20,000/well), fibroblasts (15,000/well) and endothelial cells (5,000/well) (cultured in medium containing 10% serum) were added to the wells, and the cell survival index, defined as fluorescence in percent of control cultures, was calculated after a 24, 48, and 72 h incubation period. IC_50 _values were determined as CHS 828 concentrations with a survival index below 50%.

### Cell morphology and cell death *in vitro*

Morphological changes in NB cells due to exposure to CHS 828 were assessed by phase-contrast microscopy. IMR-32 (1.5 × 10^5^/ml) were allowed to set overnight before replacing the medium with fresh medium containing 1 nM CHS 828. The cell morphology was recorded after 0, 4, 24, 48, 72, and 96 h with a digital phase-contrast microscope at × 100.

Quantification of cell death was performed by propidium iodine (PI) and DAPI (4',6-diamino-2-phenylindole) staining [24]. IMR-32 cells (1.5 × 10^5^/ml) were stained with 10 μg/ml PI and DAPI after 24, 48, and 72 h exposure to 1 nM CHS 828. Disintegration of the plasma membrane results in red fluorescence, which is a marker of cell death (determined by evaluation of at least 2,000 cells per well by UV microscopy).

### Animals

Female SCID mice (B&M, Ry, Denmark) were xenografted at the age of 6 weeks (mean body weight, 17.3 g). The animals were housed in an isolated room at 24°C with a 12-h day/night cycle. They were fed *ad libitum *with water and food pellets. Animal weight and general appearance were recorded daily throughout the experiment. The experiment was approved by the regional ethics committee for animal research.

### Xenografting and confirmation of tumor establishment

Subconfluent IMR-32 cells were harvested and kept on ice until xenotransplantation. The recipient mice were shaved and cleansed with 70% ethanol at the site of incision and anesthetized with 2% Fluothane (Zeneca Ltd., Macclesfield, UK) supplemented with 50% N_2_O in oxygen. IMR-32 cells (20 μl; 2 × 10^6 ^cells) were injected into the left adrenal gland through a left flank incision, which was closed with interrupted sutures in 2 layers. Buprenorphine (10 μg/kg; s.c.; Schering-Plough Europe, Brussels, Belgium) was administered once as postoperative analgesia. All handling of the animals was performed under aseptic conditions.

Nine weeks after xenografting, all animals (n = 35) showed establishment of primary adrenal gland tumors which was verified by re-laparotomy. Tumor volume (mean volume: 0.77 ml), was estimated as described in [25].

### Measurement of tumor volume, drug administration, perfusion fixation, and autopsy

Mice were randomized to 1 of the 3 groups: controls (peanut oil, daily, p.o., 10 days; n = 10) and CHS 828 treatment (20 mg/kg, daily, p.o.) for 10 (n = 13) or 30 days (n = 10). Administration of 20 mg/kg/day has previously been shown to be non-toxic to mice. At the study endpoints, animals were subjected to perfusion fixation [17]. After perfusion fixation, the tumors were dissected out, and their absolute weights and volumes were recorded. The internal organs were examined for macroscopic metastases (see below).

### Chromogranin A analyses

Chromogranin A (CgA) serum levels were analyzed as a marker for tumor burden and treatment efficacy. Venous blood was drawn from the right atrium before perfusion fixation. The blood was stored at 4°C overnight and spun at 135 × *g *for 10 min. The serum was removed and stored at -20°C. Serum levels of human CgA were measured by a commercial radioimmunoassay (Eurodiagnostica, Malmö, Sweden) according to the manufacturer's instructions. Only tumor-derived CgA was detected since the assay distinguishes between human and murine CgA.

### Tissue analyses

At autopsy, the organs were examined for macroscopic metastases, sliced in ~1-mm sections, and examined with a dissection microscope (× 20). Orthotopic tumors, the iliac crest, and organ biopsies with suspected metastases were dehydrated and embedded in paraffin. Tissue sections were cut at 3 μm, placed on diaminoalkyl-silane-treated glass slides, dewaxed, rehydrated, and stained immunohistochemically as described below. All these steps were performed in humid chambers at room temperature, unless otherwise indicated. After immunohistochemistry, the sections were counterstained with Harris' hematoxylin and mounted with Kaiser's glycerol gelatin (Merck).

For the quantification of angiogenesis, Bandeiraea simplicifolia-1 (BS-1) lectin was used to mark endothelial cells [25]. BS-1 (L3759; Sigma) was used at 1:50 dilution, and the sections were incubated for 2 h. Endothelial cells were used as positive controls, and the omission of the neuraminidase solution served as a negative control.

Immunohistochemical staining for DNA strand breaks (i.e. cell death) was performed by the TUNEL assay using an "*In Situ *Cell Death Detection Kit, POD" (Roche, Indianapolis, IN) according to the manufacturer's instructions. Murine ileum was used as a positive control, and the replacement of TdT with water served as a negative control.

Apoptosis was detected by staining for cleaved caspase-3 [6]. Sections were developed using Vector^® ^NovaRED™ (SK-4800, Vector Laboratories, Inc., Burlingame, CA). Human tonsil or murine colon served as a positive control, and the omission of the primary antibody served as a negative control.

Staining specific for neuroendocrine and adrenergic cells, i.e. NB cells, was performed by CgA immunohistochemistry. Before dehydration and embedding in paraffin, iliac crest biopsies were decalcified in Parengy's decalcification solution (University Hospital Pharmacy, Uppsala, Sweden) for 1 week. Tissue sections on glass slides were treated with Target Retrieval Solution (S3308, Dako) and blocked in 0.3% H_2_O_2 _for 30 min and in 1% BSA and 10% rabbit serum for 20 min. Primary antibody (M0869, Dako) was applied at 1:100 dilution for 30 min. The biotinylated secondary antibody (K335, Dako A/S) was applied at 1:80 dilution for 30 min. For detection, ABC/HRP (K355, Dako) was applied at 1:100 dilution for 30 min. The sections were developed using DAB (SK-4100, Vector). NB cell pellets were used as positive controls, and the omission of the primary antibody served as a negative control. To detect NB cells in the bone marrow of the iliac crest, 3 CgA-stained sections were examined in a blinded fashion by 2 independent investigators. Two to 3 CgA-positive cells in one section were classified as metastasis.

### Stereologic quantification

All sections were quantified at × 400 magnification in a blinded fashion [5,26]. Vascular parameters from up to 35 grids, depending on tumor size, were quantified for each tumor. Only stereologic estimates from grids with a viable upper right corner and in which the entire grid covered tumor tissue were used for quantification. If more than 50% of the upper right corner covered densely packed nuclei with sparse cytoplasm (i.e. NB cells), the grid was assigned 'viable'.

The percentage of TUNEL- and caspase-3-positive cells was calculated among ~2,000 cells in each tumor by using the upper right quarter of the counting grid mentioned above.

Treatment-related bone marrow toxicity was investigated in hematoxylin-eosin stained sections of the iliac crest. The percentage of megakaryocytes was calculated among at least 2,000 bone marrow cells.

### Statistical methods

All the data were processed in GraphPad Prism 4 for Windows (GraphPad Software Inc.). Differences between tumor volumes were analyzed with Mann-Whitney *U *test and differences in organ weight were analyzed using the Kruskal-Wallis test. Statistical differences between metastases in CHS 828-treated animals and control animals were analyzed using Fisher's exact test. Differences with p < 0.05 were considered statistically significant.

## Results

### CHS 828 is toxic to NB cells but not to fibroblasts *in vitro*

CHS 828 was more toxic to NB cells than to endothelial cells or fibroblasts *in vitro*. IC_50 _values for fibroblasts were above 10 μM CHS 828 (the highest concentration tested). Drug activity was time dependent with the first signs of toxicity after 48 h and high NB cell-specific toxicity after 72 h of continuous drug exposure (Table 1).

**Table 1:** CHS 828 toxicity profile

		IC_50_	
	24 h	48 h	72 h
htertBCE	>10 μM	200 – 500 nM	50 – 100 nM
SH-SY5Y	>10 μM	>10 μM	2 – 5 nM
IMR-32	>10 μM	>10 μM	0.2 – 0.5 nM
SK-N-SH	>10 μM	>10 μM	2 – 5 nM
CCD-1064SK	*n.d*.	*n.d*.	>10 μM

Triplicates of the NB cell lines IMR-32, SH-SY5Y, SK-N-SH cells (1 × 10^5^/ml), human foreskin fibroblasts CCD-1064SK (7.5 × 10^4^/ml) and endothelial cells htertBCE (2.5 × 10^4^/ml) were incubated with 16 different concentrations of CHS 828 for 24, 48 and 72 h (concentration range: 0.1 nM – 10 μM). Cell survival was measured by FMCA. Survival index was calculated as the percentage of viable cells at the actual concentration divided by percentage of viable cells in wells incubated without drug. Concentration intervals for IC_50_.

**Figure 1:**
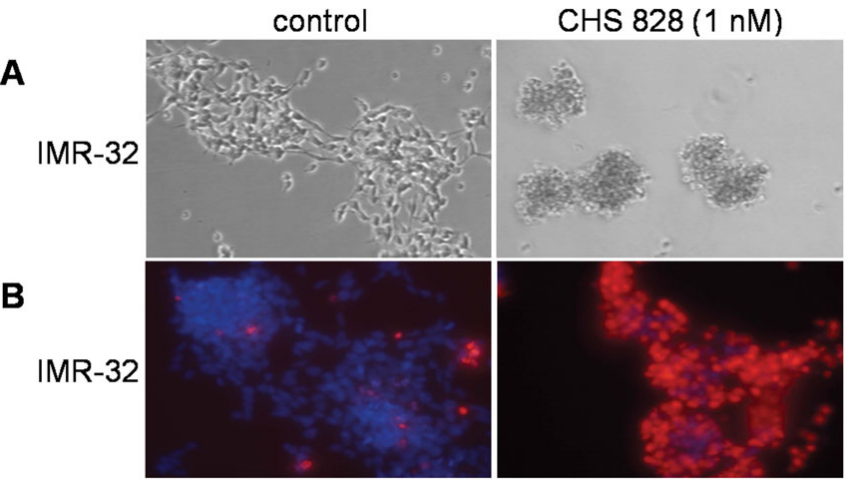
***In vitro *morphology of NB cells cultured with or without CHS 828**. IMR-32 (1.5 × 10^5^/ml) were cultured in 24-well plates in the absence (control) or presence of 1 nM CHS 828 for 0 to 72 h. Cell morphology, investigated by phase-contrast microscopy, revealed signs of cell death in NB cells exposed to CHS 828 (**A**). Viability of NB cells (IMR-32) was quantified in DAPI (4',6-diamino-2-phenylindole)-propidium iodine (10 μg/ml)-stained cells by fluorescence microscopy (**B**). Cells with intact plasma membrane (blue; DAPI staining) and cells with disrupted membrane (red; propidium iodine staining), magnification in **A**-**B**: × 100.

IMR-32 viability remained unaffected during the first 48 h of exposure to 1 nM CHS 828 but showed a 560% increase in cell death after 72 h of exposure as compared to controls (Figure 1A, B).

### CHS 828 induces regression of rapidly growing orthotopic NB *in vivo*

**Figure 2:**
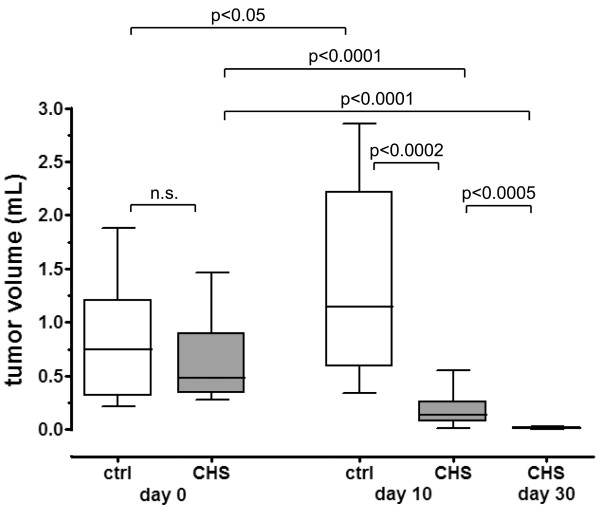
**Orthotopic NB growth in SCID mice**. SCID mice carrying orthotopic NB xenotransplants were randomized at an estimated tumor volume of 0.8 ml (□ n = 10, controls; ■ n = 23, for CHS 828 treatment). After randomization, mice were treated daily with either vehicle (□ n = 9; 10 days) or with CHS 828 (20 mg/kg; p.o.) for 10 (■ n = 13) or 30 (■ n = 10) days. Mann-Whitney *U *test was used to evaluate differences between the groups.

**Figure 3:**
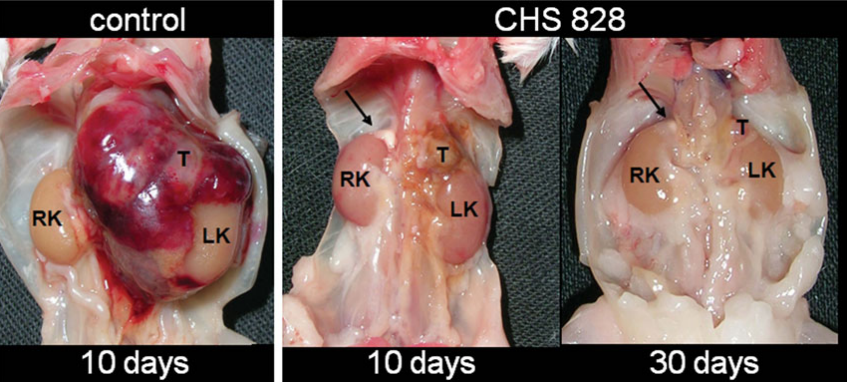
**Orthotopic NB tumors at autopsy after treatment with CHS 828 or vehicle**. Orthotopic tumors at autopsy treated with vehicle or CHS 828 (20 mg/kg/day) for 10 or 30 days. Note the brown color of the tumor after 10 days of treatment, indicating areas of resorbed hemorrhage. T = tumor, RK = right kidney, LK = left kidney; arrows indicate the normal right adrenal gland.

Tumors from vehicle-treated animals grew significantly within 10 days from randomization (p < 0.05) (Figure 2, Figure 3). Despite this rapid growth, no tumor rupture or intraperitoneal bleeding was observed. Daily treatment with CHS 828 (20 mg/kg; p.o.) for 10 days significantly reduced mean tumor volume (-89%) and weight (-92%) compared to untreated littermates (p = 0.0002 and p = 0.0001, respectively). An additional 20 days of treatment (total of 30 days) further reduced tumor volume (-92%) and weight (-86%) compared to short term treatment (p = 0.0005 and p = 0.0006, respectively) (Figure 2, Figure 3). Administration of CHS 828 resulted in tumor regression (final tumor volume compared to starting volume) after 10 (-81%; p < 0.0001) and 30 days (-98%; p < 0.0001). A detailed summary of tumor data is provided in Additional file 1 (see Additional file 1: Observation parameters of tumor-bearing SCID mice during the experiment).

In addition to the reduction in tumor volume, treatment with CHS 828 for 10 days also significantly reduced the percentage of viable tumor tissue from 75.5% to 15.4% (p < 0.0001) and increased the fraction of dead (i.e. TUNEL positive) cells from 26.8% to 78.2% (p < 0.0001). The fraction of apoptotic cells was not different compared to controls when quantified by caspase-3 immunohistochemistry.

There were no adverse effects of CHS 828 on the general status of the animals. CHS 828 did not affect the body or organ weight (liver, spleen, lung and kidney) in any of the treated animals compared with controls (see Additional file 2: Organ weight of healthy and tumor-bearing SCID mice). Furthermore, no treatment-related diarrhea or vomiting was observed, and the percentage of megakaryocytes in the bone marrow of the iliac crest did not differ between treated and healthy animals (2.46% ± 0.36% and 2.57% ± 0.40%, respectively; n.s.). Three mice were excluded from the study: 2 mice before (1 due to inexplicable weight loss and 1 due to paraplegia) and 1 mouse after randomization (paraplegia; control group). The 2 cases of paraplegia were caused by orthotopic NB growth extending into the spinal canal.

### Metastatic pattern of orthotopic NB mimics disseminated disease in high-risk NB patients

Few large, macroscopic organ metastases were observed at autopsy. Examination of the lung, liver, spleen, bone marrow, and both kidneys under a dissection microscope revealed NB spread to many of these organs. This was confirmed by either hematoxylin-eosin staining or CgA immunohistochemistry. Table 2 summarizes NB spread in this orthotopic model compared to clinical NB.

**Table 2:** Invasive pattern of orthotopic NB in SCID mice

		Orthotopic mouse model			Clinical NB
Site		Control at 10 days	CHS 828 at 10 days	CHS 828 at 30 days	[28]

Animals with metastases		100% (9/9)	46% (6/13)*	40% (4/10)*	

Lung		11% (1/9)	0% (0/13)	0% (0/10)	34%
Liver		78% (7/9)	23% (3/13)*	0% (0/10)***	30%
Spleen		22% (2/9)	8% (1/13)	30% (3/10)	n.d.
Bone marrow	iliac crest spine^a^	78% (7/9) 22% (2/9)	23% (3/13)* 8% (1/13)	10% (1/10)** 10% (1/10)	71%
Bone		n.d.	n.d.	n.d.	56%
Lymph nodes		n.d.	n.d.	n.d.	31%
Kidney invasion^†^		22% (2/9)	0% (0/13)	0% (0/10)	n.d.

Metastatic spread in SCID mice carrying orthotopic NB xenotransplants treated either with CHS 828 (20 mg/kg/day) or vehicle compared to metastatic incidence of NB at INSS stages 4 and 4S in clinic [28]. Data shows microscopic metastases in the marrow of the iliac crest, and composite data of macro- and microscopic metastases to the organs.

* < 0.05; ** < 0.01; *** < 0.001 (compared with controls); Fisher's exact test.

^a^NB cells in the spine and kidney were regarded as continuous tumor growth.

n.d., not determined; NB, neuroblastoma; INSS, International Neuroblastoma Staging System

**Figure 4:**
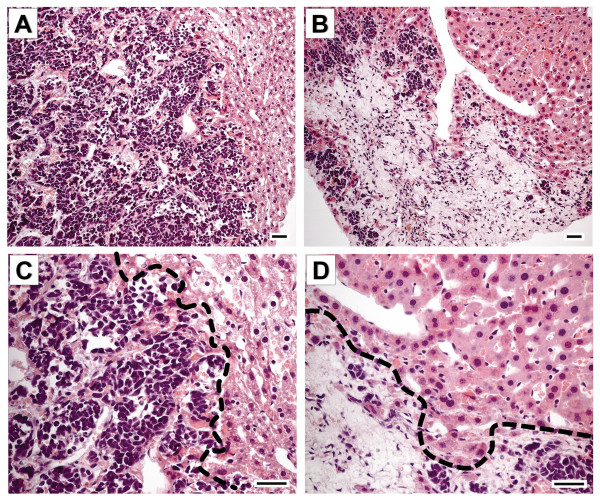
**Metastatic NB growth in the liver**. Liver metastases were smaller and exhibited large necrotic areas after 10 days of CHS 828 treatment (**B**) compared to controls (**A**). "**C**" and "**D**" are magnifications of "**A**" and "**B**", respectively. In **C **and **D **the border between healthy liver tissue and either viable tumor tissue (densely packed nuclei with sparse cytoplasm) (**C**) or areas of tumor necrosis (**D**) is outlined. Hematoxylin-eosin staining; bars = 20 μm.

The frequency of NB spread was reduced by CHS 828 compared with controls. Postmortem classification according to the INSS (International Neuroblastoma Staging System) showed that all control animals were classified as stage 4. Metastases detected in the treatment groups were smaller and showed morphological signs of regression (tumor necrosis) compared with metastases detected in controls (Figure 4).

In 2 control animals, there was NB growth in the thymus, thoracic lymph nodes, and along the thoracic vertebrae, whereas no NB spread to these sites could be detected in CHS 828-treated animals. Postmortem evaluation of treatment efficiency by applying the INSS revealed a trend toward lower stages (better resectability) when tumors were treated with CHS 828 (Table 3). No peritoneal metastases were detected, indicating that no free tumor cells were seeded onto the peritoneal surface during xenotransplantation.

**Table 3:** Staging of orthotopic NB in SCID mice

		Orthotopic mouse model		
		Control at 10 days	CHS 828 at 10 days	CHS 828 at 30 days

INSS	stage 1	0% (0/9)	46% (6/13)	60% (6/10)
	stage 2^a^	0% (0/9)	8% (1/13)	0% (0/10)
	stage 3	0% (0/9)	0% (0/13)	0% (0/10)
	stage 4^b^	100% (9/9)	46% (6/13)	40% (4/10)

Postmortem classification of control and CHS 828-treated animals using the INSS criteria for staging [27].

^a^No extensive lymph node investigation was performed; therefore, "stage 2" was not divided into "2A" and "2B"

^b^INSS stage 4 was not separated into stages 4 and 4S since 4S is for infants

INSS (International Neuroblastoma Staging System) according to Simpson and Gaze [27] at autopsy

### Reduced CgA-levels in serum of CHS 828-treated animals

Human CgA was detected in the serum of all vehicle-treated controls (n = 9) (10.7 ± 4.0 nmol/L). However, in 10 days study with CHS 828, only 1/13 mice showed a detectable concentration of CgA (1 nM/L), and no animals receiving long-term treatment (n = 10) or healthy littermates without tumors (n = 5) had detectable CgA concentrations in serum (detection limit: 0.8 nmol/L) (p < 0.001).

### CHS 828 reduces tumor angiogenesis

**Figure 5:**
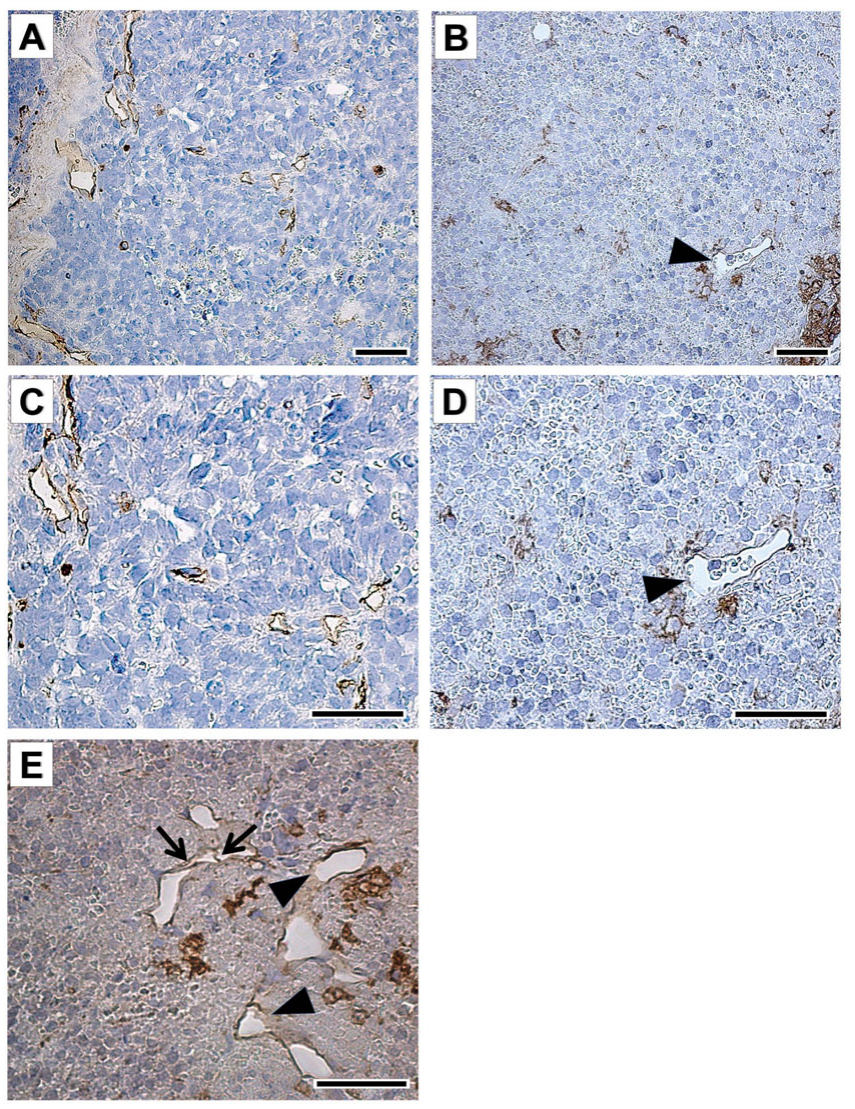
**Representative morphology and vessel profile in orthotopic NB xenografts**. Vehicle-treated tumors (control) contained a larger number of small vessels (stained in brown) (**A, C**) compared to CHS 828 (20 mg/kg/day; p.o.) treated tumors (**B, D-E**) already 10 days after randomization. Vessels of control tumors had a thin endothelial cell lining (brown) (**A, C**). CHS 828 treated tumors revealed vessels only partly surrounded by endothelial cells (arrowheads) (**B, D-E**) or endothelial cells detaching from the basement membrane (arrows) (**E**). **C **and **D **are magnifications of **A **and **B**, respectively. Bandeiraea simplicifolia-1 (BS-1) lectin staining (brown); bar = 40 μm.

Daily administration of CHS 828 altered vascular parameters as determined by stereology (Table 4). Vessel density, vessel length density (L_v_), and surface density (S_v_) were significantly reduced in these tumors compared to vehicle-treated controls. The vessel volumetric density (V_v_) was reduced in CHS 828-treated tumors but the reduction was not significant (p = 0.09) (Table 4). A single layer of endothelial cells encircled the lumen of vessels in untreated tumors (Figure 5A and Figure 5C) whereas in CHS 828 treated tumors, endothelial cells were frequently not entirely surrounding the lumen (Figure 5B, D, E) or detaching from the basement membrane (Figure 5E). Despite the incomplete endothelial cell lining, only 1/13 (8%) of the animals treated with CHS 828 for 10 days showed intra-tumor hemorrhage, defined as erythrocytes outside vessel lumen, whereas 9/9 (100%) of the tumors in control animals had erythrocytes in the tumor tissue.

**Table 4:** Quantification of tumor angiogenesis by stereology

	**vessel density** (mm^-2^)	**Lv** (mm^-2^)	**Vv** (10^-3^)	**Sv** (mm^-1^)
**control **(n = 9)	39.9 ± 18.5	77.2 ± 37.1	5.1 ± 2.5	2.6 ± 1.3
**CHS 828^a ^**(n = 13)	12.8 ± 10.3	25.6 ± 20.5	2.8 ± 2.1	1.0 ± 0.7
*Change*^b^*(%)*	-67.1% **	-67.1% **	*-44.7%*	-62.7% *

CHS 828 was administered at 20 mg/kg/day by oral gavage.

Lv, length of vessels per tumor volume (length density); Vv, volume of vessels per tumor volume (volumetric density); Sv, surface area of vessels per tumor volume (surface density). Mean ± 1SD, Mann-Whitney *U *test.

^a^CHS 828 treatment for 10 days

^b^Change compared to control

*p < 0.05 **p < 0.01

## Discussion

In this study, we developed an orthotopic model for high-risk NB and characterized tumor spread in this model. Our results showed that orthotopic implantation of MYCN amplified NB cells into the adrenal gland favors metastatic spread since all the control animals developed macroscopic metastasis. Postmortem NB staging according to the INSS criteria was performed to address the metastatic pattern of NB [27]; the result showed that the metastatic pattern of MYCN-amplified NB cells in this model resembled high-risk NB. However, we observed a higher incidence of liver metastases in our model as compared to children with INSS stage 4 and older than 1 year. A possible explanation is the MYCN amplification status of the NB cells (IMR-32) used for orthotopic xenotransplantation in this study. MYCN amplification in NB increases risk for tumor spread to the liver, which in turn significantly decreases 3 year event-free survival in the patient group of INSS stage 4 and age over 1 year [28].

Using this orthotopic model for high-risk NB, we examined the effect of daily administration of the cyanoguanidine CHS 828 (20 mg/kg/day; equal to 60 mg/m^2^/day) on the growth and metastatic potential of this highly malignant neuroendocrine tumor. The dose chosen is considered low since the lethal dose mice has been shown to be 853 mg/m^2 ^and MTD in phase I studies was 228 mg/m^2 ^[18]. The dose is also lower when compared to another preclinical study where CHS 828 was administered to mice at 100 mg/kg/week (300 mg/m^2^/week) and 250 mg/kg/week (750 mg/m^2^/week) (designated "low" and "high" dose, respectively) [14]. Interestingly, the 300 mg/m^2^/week dose only reduced neuroendocrine tumor growth.

In our study we showed that CHS 828 induced tumor regression, reduced the viable tumor tissue fraction, and reduced the number of animals with metastases and number of metastases per animal without causing toxicity. This finding is of considerable importance since CHS 828 successfully treated large, established tumors that were more than twice the size of s.c. tumors in the study of Svensson *et al*. [17]. Additionally, we observed tumor regression whereas s.c. tumors showed reduced growth compared to controls [17]. The more pronounced treatment efficacy in the metastatic model mimicking clinical disseminated disease compared to heterotopic, s.c. models indicates that orthotopic models should be considered in preclinical drug screening programs.

Postmortem staging of treated animals showed a trend toward lower INSS stages compared to controls. In addition to tumor staging, we investigated the potential value of CgA serum levels for predicting treatment outcome. CgA is an acidic, monomeric protein and is co-stored and co-released with catecholamines from secretory granules in neural, endocrine, and neuroendocrine cells [29]. CgA was almost exclusively detected in serum from INSS stage 4 mice. Thus, our results support the concept of NB as a neuroendocrine tumor and the suitability of CgA as a NB tumor marker [30-32] and as an indicator of treatment efficacy.

Immunohistological studies of tumor sections showed morphological signs of cell death, i.e. condensed and fragmented nuclei, after CHS 828 treatment for 10 days, causing a reduction of the viable tumor fraction by more than a factor of 5.7 compared to controls. The decrease in viable tissue fraction was independent of activated caspases-3. This observation is supported by studies reporting that CHS 828 induces late programmed cell death with features not related to classical apoptosis [33,34]. In fact, CHS 828 has been reported to inhibit cellular synthesis of NAD resulting in energy depletion, and subsequent cell death [12]. NAD is produced primarily through biochemical salvage pathway using nicotinamide as a substrate. CHS 828 inhibits NAD synthesis from nicotinamide only after continuous and long time exposure [12]. Delayed cell death was confirmed in our *in vitro *studies in which the viability of human NB cells (IMR-32, SK-N-SH and SH-SY5Y) was affected only after prolonged exposure to CHS 828.

CHS 828 caused cell death in all three NB cell lines *in vitro *with IC_50 _values 20 × below values of endothelial cells. Human fibroblasts never reached IC_50 _values at concentrations tested (0.1 nM – 10 μM).

Compared to results from Åleskog *et al*. who tested CHS 828 toxicity on human lymphocytes in the same FMCA protocol described here, NB cells in our study had lower IC_50 _values [35]. This indicates a higher drug sensitivity of NB cells. We speculate that the high CHS 828 sensitivity of the NB cell lines might be due to an active uptake of CHS 828 in NB cells, mediated by the noradrenalin transport transmembrane protein in analogy with MIBG [36]. It has been shown that the human NB cells used in this study are so-called MIBG-positive cell lines (a characteristic shared with 85% of NB cells in patients) in which there is an apparent noradrenalin transporter gene expression [37,38]. MIBG is a molecule that is specifically taken up by most NB cells [39] and cytotoxic drugs with structural homology to MIBG (e.g. CHS 828) may have a similar selectively for NB cells. To address the question whether CHS 828 was less active in cell lines with greater avidity for MIBG, we included the NB cell line SK-N-SH in our *in vitro *toxicity studies. CHS caused cell death in all NB cell lines without any correlation to their avidity in taking up MIBG. We therefore conclude that CHS 828 could be taken up by different NB cells despite presence of chlorophenoxyhexyl and cyano groups in the chemical structure of this drug.

As rodents have been shown to tolerate higher CHS 828 levels than man both *in vitro *[40] and *in vivo *[41], the dose chosen in the current study can be considered low for the host cells (including the endothelial cells) but higher for the human tumor cells. Despite this, both tumor vessels of murine origin and human tumor cells were affected by treatment with CHS 828. Thus, we believe that the current administration of CHS 828 represents a dual targeting approach involving the inhibition of angiogenesis, and direct tumor cell toxicity. The two processes (angiogenesis inhibition and tumor cell toxicity) may have different kinetics and may vary in proportion with the distance from the nearest vessel. Furthermore, treated animals showed less intra-tumor hemorrhage than controls. Therefore, the vasculature in tumors treated with CHS 828 was more stable than vessels in rapidly growing, untreated tumors indicating vessel normalization [42].

More prolonged schedules of CHS 828 have previously been shown to increase antitumor activity as well as toxicity *in vitro *[13], *in vivo *[41] and clinically [18,19]. In the current study, no bone marrow toxicity due to prolonged exposure to low doses of CHS 828 was found. This was investigated by quantifying the percentage of megakaryocytes in the bone marrow of the iliac crest. Megakaryocytes, the precursors of platelets, were easily identified despite the disorderly arranged cells in the bone marrow. We found that the frequency of megakaryocytes in the bone marrow was not affected by CHS 828 treatment.

In clinical phase I studies, CHS 828 showed a large variation in drug uptake both between and within patients [18,19] which was also observed in a previous study in nude mice [17]. This inter-individual variability has partly been explained by variations of hepatic and intestinal CYP3A4 activity, an enzyme important for metabolizing cyanoguanidines such as CHS 828 [19,43]. Another explanation for this variability might be related to the low solubility of CHS 828 hampering uptake in the gastrointestinal tract. Clinical trials using orally administered CHS 828 were discontinued due to the variation in exposure levels and dose limiting toxicities. Hence a water soluble prodrug, EB1627 (GMX1777) was synthesized by adding a tetraethylenglycol moiety to the parent drug CHS 828 (GMX1778). This compound could be administered i.v. thus allowing a controlled dosing to the patient. After intravenous administration, the tetraethylenglycol moiety rapidly dissociates and releases CHS 828 without reducing antitumor activity [44].

## Conclusion

We believe that the metastatic and clinically relevant model evaluated here provides an excellent tool for examining new treatment strategies in children with high-risk NB. Based on data derived from this model, we suggest that the active compound CHS 828 might provide clinical benefits in treating children with high-risk NB.

## Competing interests

The authors declare that they have no competing interests.

## Authors' contributions

DF, RC and FA designed the study. DF acquired data which was analyzed by DF and FA, except for CgA data which was analyzed by MS. RC contributed with data interpretation and drafting of the manuscript written by DF and FA. EL and MS provided input in writing of the manuscript. All authors read and approved the manuscript.

## Supplementary Material

Additional File 1 **Observation parameters of tumor-bearing SCID mice during the experiment**. A table summarizing individual follow-up of body weight and tumor development for each individual mouse in the study. Statistical analysis (Mann-Whitney *U *test) indicates group differences in tumor volume, tumor weight and tumor index (tumor weight/final body weight × 100). Click here for file

Additional File 2 **Organ weight of healthy and tumor-bearing SCID mice**. A table summarizing organ weight for each individual mouse in the study, including healthy littermates. Statistical analysis (Kruskal Wallis test). Click here for file
