# Supplementary material for: Regression of orthotopic neuroblastoma in mice by targeting the endothelial and tumor cell compartments
Source: J Transl Med. 2009 Mar 12;7:16. doi: 10.1186/1479-5876-7-16 (PMC2667491; doi:10.1186/1479-5876-7-16)
Supplement: Additional File 1 — Observation parameters of tumor-bearing SCID mice during the experiment. A table summarizing individual follow-up of body weight and tumor development for each individual mouse in the study. Statistical analysis (Mann-Whitney U test) indicates group differences in tumor volume, tumor weight and tumor index (tumor weight/final body weight × 100). [file 1479-5876-7-16-S1.doc]

Additional file 1: Observation parameters of tumor-bearing SCID mice during the experiment

| Control | | | | | | |  | CHS 828 | | | | | | |
| --- | --- | --- | --- | --- | --- | --- | --- | --- | --- | --- | --- | --- | --- | --- |
| SCID mouse number | body weight (g)  start final | | tumor weight (g) | tumor volume (mL)  start final | | tumor index^a^ (%) |  | SCID mouse number | body weight (g)  start final | | tumor weight (g) | tumor volume (mL)  start final | | tumor index^a^ (%) |
|  |
| 10 days | |  |  |  |  |  |  | 10 days of treatment | | |  |  |  |  |
| 1 | 18.4 | 25.2 | 2.882 | 1.466 | 1.683 | 11.4 |  | 1 | 15.6 | 19.4 | 0.123 | 0.745 | 0.114 | 0.6 |
| 2 | 16.1 | 21.9 | 1.164 | 0.484 | 1.147 | 5.3 |  | 2 | 18.5 | 21.5 | 0.332 | 1.413 | 0.412 | 1.5 |
| 3 | 17.6 | 22.5 | 2.225 | 0.392 | 2.067 | 9.9 |  | 3 | 19.0 | 21.9 | 0.135 | 0.616 | 0.054 | 0.6 |
| 4 | 16.3 | 21.6 | 2.345 | 0.760 | 2.368 | 10.9 |  | 4 | 16.6 | 19.6 | 0.078 | 1.014 | 0.135 | 0.4 |
| 5 | 18.1 | 22.1 | 1.764 | 0.799 | 2.857 | 8.0 |  | 5 | 14.7 | 18.2 | 0.109 | 1.881 | 0.123 | 0.6 |
| 6 | 16.4 | 20.5 | 0.871 | 0.282 | 0.849 | 4.2 |  | 6 | 19.0 | 22.2 | 0.138 | 0.338 | 0.087 | 0.6 |
| 7 | 16.7 | 19.0 | 0.491 | 0.321 | 0.344 | 2.6 |  | 7 | 18.0 | 20.7 | 0.126 | 1.204 | 0.169 | 0.6 |
| 8 | 17.2 | 20.3 | 0.993 | 0.356 | 0.892 | 4.9 |  | 8 | 19.1 | 21.8 | 0.010 | 1.294 | 0.018 | 0.0 |
| 9 | 17.6 | 21.7 | 2.022 | 1.000 | 0.659 | 9.3 |  | 9 | 17.6 | 22.3 | 0.271 | 0.905 | 0.358 | 1.2 |
|  |  |  |  |  |  |  |  | 10 | 17.6 | 22.0 | 0.301 | 1.639 | 0.278 | 1.4 |
|  |  |  |  |  |  |  |  | 11 | 16.6 | 19.6 | 0.103 | 0.905 | 0.235 | 0.5 |
|  |  |  |  |  |  |  |  | 12 | 18.4 | 21.0 | 0.030 | 0.310 | 0.011 | 0.1 |
|  |  |  |  |  |  |  |  | 13 | 15.7 | 17.4 | 0.047 | 0.704 | 0.053 | 0.3 |
| mean values after 10 days of treatment | | | | | | |  | 30 days of treatment | | |  |  |  |  |
|  | body weight | | tumor | tumor volume | | tumor |  | 14 | 16.5 | 18.8 | 0.003 | 0.338 | 0.006 | 0.0 |
|  | (g) | | weight | (mL) | | index^a^ |  | 15 | 17.5 | 21.2 | 0.051 | 1.782 | 0.018 | 0.2 |
|  | start | final | (g) | start | final | (%) |  | 16 | 16.2 | 21.6 | 0.043 | 0.310 | 0.001 | 0.2 |
| control | 17.2 | 21.6 | 1.640 | 0.653 | 1.430 | 7.4 |  | 17 | 16.5 | 19.5 | 0.013 | 0.310 | 0.011 | 0.1 |
| CHS 10d | 17.3 | 20.6 | 0.139* | 0.820 | 0.157* | 0.7* |  | 18 | 18.3 | 21.1 | 0.007 | 0.259 | 0.006 | 0.0 |
| CHS 30d |  | 20.6 | 0.020** |  | 0.013** | 0.1** |  | 19 | 17.6 | 22.0 | 0.042 | 0.484 | 0.033 | 0.2 |
| *p < 0.001 compared to control (Mann-Whitney *U* test) | | | | | | |  | 20 | 17.3 | 20.0 | 0.002 | 0.237 | 0.005 | 0.0 |
| **p < 0.001 compared to 10 days of treatment (Mann-Whitney *U* test) | | | | | | |  | 21 | 16.7 | 20.0 | 0.009 | 1.207 | 0.015 | 0.0 |
| ^a^tumor in % body weight | | | | | | |  | 22 | 18.2 | 21.7 | 0.027 | 0.748 | 0.024 | 0.1 |
| (tumor weight/final body weight) ×100 | | | | | | |  | 23 | 17.5 | 20.0 | 0.000 | 0.216 | 0.012 | 0.0 |
