# Supplementary material for: Regression of orthotopic neuroblastoma in mice by targeting the endothelial and tumor cell compartments
Source: J Transl Med. 2009 Mar 12;7:16. doi: 10.1186/1479-5876-7-16 (PMC2667491; doi:10.1186/1479-5876-7-16)
Supplement: Additional File 2 — Organ weight of healthy and tumor-bearing SCID mice. A table summarizing organ weight for each individual mouse in the study, including healthy littermates. Statistical analysis (Kruskal Wallis test). [file 1479-5876-7-16-S2.doc]

Additional file 2: Organ weight of healthy and tumor-bearing SCID mice

| Control |  |  |  |  |  | CHS 828 | |  |  |  |
| --- | --- | --- | --- | --- | --- | --- | --- | --- | --- | --- |
| SCID mouse number | kidney (g) | lungs (g) | spleen (g) | liver (g) |  | SCID mouse number | kidney (g) | lungs (g) | spleen (g) | liver (g) |
|  |
| healthy littermates | | |  |  |  | 10 days of treatment | | |  |  |
| a | 0.201 | 0.251 | 0.048 | 1.328 |  | 1 | 0.181 | 0.325 | 0.090 | 1.413 |
| b | 0.171 | 0.255 | 0.043 | 1.362 |  | 2 | 0.194 | 0.265 | 0.109 | 1.968 |
| c | 0.195 | 0.286 | 0.056 | 1.026 |  | 3 | 0.188 | 0.268 | 0.084 | 1.771 |
| d | 0.188 | 0.330 | 0.068 | 1.888 |  | 4 | 0.198 | 0.244 | 0.060 | 1.349 |
| e | 0.214 | 0.252 | 0.068 | 1.850 |  | 5 | 0.185 | 0.220 | 0.047 | 1.247 |
| controls 10 days | | |  |  |  | 6 | 0.203 | 0.300 | 0.076 | 1.657 |
| 1 | 0.225 | 0.238 | 0.142 | 1.877 |  | 7 | 0.164 | 0.251 | 0.067 | 1.318 |
| 2 | 0.170 | 0.240 | 0.101 | 1.574 |  | 8 | 0.218 | 0.275 | 0.058 | 1.544 |
| 3 | 0.163 | 0.177 | 0.071 | 1.282 |  | 9 | 0.212 | 0.442 | n.d. | 2.897 |
| 4 | 0.210 | 0.258 | 0.142 | 1.742 |  | 10 | 0.219 | 0.265 | 0.072 | 1.889 |
| 5 | 0.233 | 0.298 | 0.116 | 1.659 |  | 11 | 0.215 | 0.271 | 0.072 | 1.393 |
| 6 | 0.172 | 0.232 | 0.080 | 1.316 |  | 12 | 0.191 | 0.232 | 0.077 | 1.960 |
| 7 | 0.168 | 0.181 | 0.041 | 1.174 |  | 13 | 0.150 | 0.223 | 0.059 | 1.195 |
| 8 | 0.169 | 0.227 | 0.054 | 1.695 |  | 30 days of treatment | | |  |  |
| 9 | 0.206 | 0.227 | 0.117 | 1.880 |  | 14 | 0.176 | 0.315 | 0.046 | 1.608 |
|  |  |  |  |  |  | 15 | 0.198 | 0.266 | 0.074 | 1.467 |
|  |  |  |  |  |  | 16 | 0.172 | 0.263 | 0.088 | 1.341 |
|  | | | |  |  | 17 | 0.191 | 0.266 | 0.056 | 1.435 |
| mean organ weight (g) | | | |  |  | 18 | 0.184 | 0.289 | 0.048 | 1.332 |
|  | kidney | lungs | spleen | liver |  | 19 | 0.202 | 0.265 | 0.070 | 1.770 |
| healthy | 0.194 | 0.275 | 0.057 | 1.491 |  | 20 | 0.189 | 0.228 | 0.045 | 1.397 |
| control | 0.191 | 0.231 | 0.096 | 1.578 |  | 21 | 0.165 | 0.196 | 0.048 | 0.932 |
| CHS 10d | 0.194 | 0.275 | 0.073 | 1.662 |  | 22 | 0.174 | 0.279 | 0.051 | 1.268 |
| CHS 30d | 0.185 | 0.267 | 0.060 | 1.411 |  | 23 | 0.196 | 0.304 | 0.071 | 1.561 |

n.d. not determined; differences in mean organ weight not significant; Kruskal-Wallis test
